# Examination of factors affecting insomnia in older victims of the Kumamoto earthquake

**DOI:** 10.7717/peerj.20584

**Published:** 2026-01-13

**Authors:** Yumie Kanamori, Tomonori Samiso, Ayako Ide-Okochi

**Affiliations:** 1 Graduate School of Health Sciences, Kumamoto University, Kumamoto City, Japan; 2 Health and Welfare Policy Division, Health and Welfare Bureau, Kumamoto City, Japan

**Keywords:** Earthquake, Lifestyle, Social capital, Insomnia, Older adults

## Abstract

**Objective:**

Post-disaster survivors are prone to increased risks related to mental disorders such as insomnia and depression. In addition, it has been recently noted that elderly people have difficulty falling asleep and waking up in the middle of the night, decreasing sleep efficiency. Therefore, there is a need to understand the actual situation of insomnia among the older persons affected after the earthquake and consider appropriate support. However, the actual situation after the Kumamoto earthquake in 2016 has not been clarified. Therefore, this study aimed to examine the factors affecting insomnia among the older adults affected by the Kumamoto earthquake.

**Methods:**

The study included 4,758 persons (2,010 men and 2,748 women; mean age 75.36 ± 7.33 years) aged 65 years or older among those who had moved out of temporary housing in Kumamoto City. The survey was a self-administered questionnaire sent by mail. The independence of each variable was confirmed using the *χ*^2^ test. Binomial logistic regression analysis was used to examine factors influencing insomnia.

**Results:**

Persons significantly more likely to report insomnia had the following characteristics: age ≥ 75, no exercise habits, not eating three times a day, no appetite, eating alone, not participating in community activities, not knowing information about community activities, no one to talk to, no family to talk to, and no coworkers to talk to. Conversely, those less likely to fall into the insomnia category had the following attributes: men, those who had no employers, those who consulted friends, those who consulted a medical institution, those who consulted a care welfare office, and those who consulted city hall. Additionally, the respondents were more likely to report insomnia when they moved out of the community.

**Discussion:**

Support for insomnia requires both informal supports to prevent isolation based on mutual community support and formal support for those suspected of insomnia.

## Introduction

Eight years have passed since the April 2016 Kumamoto earthquake (Kumamoto City, 2016). Due to natural disasters worldwide, health crisis management is becoming increasingly important, and post-disaster victims are at an increased risk of mental disorders such as depression (Matsubara et al., 2014) and post-traumatic stress disorder (hereinafter referred to as PTSD). They also need long-term support (Ide-Okochi et al., 2022). In addition, mental disorders such as depression increase the risk of developing further mental disorders (Li et al., 2016) as well as the appearance of insomnia from early onset.

According to a country-by-country comparison of average sleep duration released in 2021 by the Organization for Economic Cooperation and Development (OECD), Japan had the shortest sleep (7 h 22 min per day) duration among 33 countries around the world, mainly developed countries (OECD, 2021). The United States, which has the longest sleep duration among OECD member countries, had 8 h 51 min, an hour and a half longer than Japan. Therefore, Japan’s (Ministry of Health Labor Welfare, 2023) proposed the Sleep Guide for Health Promotion 2023 and indicated that it is important to ensure that both quality (sleep restfulness) and quantity (sleep duration) of sleep are adequate. In addition to daytime sleepiness, sleep problems are likely to cause increased psychosomatic complaints such as headaches, emotional instability (Vandekerckhove & Wang, 2017), and impaired attention and judgment (Groeger et al., 2014). It is also associated with an increased risk of developing or worsening symptoms of obesity (Häusler et al., 2019), hypertension (Wang et al., 2017), type 2 diabetes (Reutrakul & Van Cauter, 2018), heart disease (Korostovtseva, Bochkarev & Sviryaev, 2021), cerebrovascular disease (Chaudhry et al., 2020) and mortality (Ensrud et al., 2012).

On the other hand, economic hardship, loss of employment, and a pessimistic outlook reportedly have a significant impact on mental health transitions, including insomnia and suicidal ideation (Orui, Kuroda & Yusumura, 2019; Xu et al., 2018). Furthermore, there is evidence that the standardized mortality rate for suicide increases after survivors leave temporary housing (Orui et al., 2020). Since many depressed individuals commit suicide, it is important to focus on insomnia, which tends to develop early in the course of depression. It is also essential that insomnia be detected early and that local health care professionals provide appropriate support in post-earthquake areas, where the risk of mental disorders is likely to increase.

However, in recent years, the elderly tend to sleep longer because of reduced sleep restfulness caused by difficulty falling asleep, and reduced sleep efficiency (Yuan et al., 2022) due to mid-sleep awakenings. Prolonged sleep increases the risk of future mortality by a factor of 1.33 (Da Silva et al., 2016). Therefore, it is necessary to take measures to reduce the difficulty of falling asleep and awakening in the middle of the night, which cause long hours of sleep. Specifically, being active during the day and having a good day/night pattern contributed to good sleep (Ministry of Health Labor Welfare, 2023). Conversely, a lack of daytime activity and reduced day-night crispness can facilitate the emergence of daytime sleepiness and fatigue (Buysse et al., 2011) and lead to prolonged naps (Da Silva et al., 2016), preventing good sleep at night. In contrast, exercise helps shorten the sleep onset latency (Matsumoto et al., 2017) and improves subjective sleep quality (Hasan et al., 2022); eating breakfast prevents the body clock from going backward, contributing to sleepiness and sleep deprivation (Otsuka et al., 2023). Social capital such as community activities, interpersonal relationships, and trust (Ministry of Health Labor Welfare, 2023) also promotes good sleep and physical activity. Therefore, it is important to actively promote daytime activities such as exercise, diet, and social capital to achieve good night sleep.

Many people were forced to relocate after the disaster because of housing damage. Sugawara, Tohmata & Tsuji (2019) found that the odds ratio of insomnia was significantly higher in the group that relocated than in the group that did not and that the association with insomnia was stronger when the relocation site was further away from the district where the person had lived before the disaster. In addition, in the areas affected by the Great East Japan Earthquake, the rate of insomnia increased after people moved from temporary housing to permanent housing, which they moved into immediately after the disaster (Tsuji, 2019). Therefore, it is speculated that relocation may also affect insomnia.

Based on the above, it is necessary to understand the actual situation and factors affecting sleep-related problems among older adults affected by the disaster and to consider appropriate support after the earthquake. During the Kumamoto earthquake, over 180,000 people were affected and up to 110,000 were forced to move to temporary housing (Kumamoto City, 2016), of which approximately 24,000 (21%) were older adults. Therefore, support for insomnia among older adults affected by the Kumamoto earthquake is considered a medium- to long-term issue. However, at present, there are no findings that clarify the actual situation of insomnia and its factors. Therefore, we examined whether the extent of relocation and daytime activities such as exercise, diet, and social capital explain insomnia among older adults affected by the Kumamoto earthquake. Identifying the factors that influence insomnia will help local health and welfare professionals develop and implement measures to prevent mental disorders in older adults affected by disasters. Therefore, this study examined the factors affecting insomnia among older adults affected by the Kumamoto earthquake and to obtain suggestions for future health activities.

## Materials & Methods

### Study population

The study population were persons aged 65 years or older affected by the 2016 Kumamoto earthquake who had moved out of temporary housing by December 2019 living in Kumamoto City. The survey was conducted using a self-administered questionnaire for all those aged 18 and older. The questionnaires were mailed to 11,479 households that had moved out. Individuals from each household answered the questionnaire and returned it by mail. Of the 8,966 questionnaires collected, 4,758 persons aged 65 years or older were included. Data were collected from July to December 2020. We received written informed consent from participants of our study. In Kumamoto Prefecture, several local authorities have conducted recovery surveys. The survey conducted by Kumamoto Prefecture showed that changes in the community after the earthquake reduced opportunities for physical activity, and that relocation impacted on mental health and long-term psychology (Ide-Okochi et al., 2022). However, this study is the first to examine insomnia among elderly after an earthquake using a reconstruction survey conducted in Kumamoto City. Note that this study used the Kumamoto City Reconstruction Survey data to avoid that the subjects had to answer repeat surveys. The results of this study provide a basic data reference for individual support by public health nurses and group support at community centers. They will also be used by Kumamoto City to gain insight into community-based support measures and to design policies. Kumamoto City has a population of 73,8567 (in 2020) and is an ordinance-designated city. Among them, 196,435 are aged 65 and over, and the aging rate is approximately 27%. Kumamoto City is providing comprehensive support for reconstruction, addressing issues such as seamless livelihood reconstruction, health support, and psychological care. This study was reviewed and approved by the Ethics Committee of the Department of Epidemiology and General Medicine at the Kumamoto University Research on Life Sciences and Medical Sciences for Human Subjects (1940).

### Variables

#### Basic attributes

Regarding basic attributes, the respondents were asked about their gender, age, cohabitants, temporary housing category, and current residence. Age was measured using a descriptive form in which the respondents were asked to state their current age. Gender was linked to the Kumamoto City ledger. Question: Do you have anyone living with you? The respondents were asked to answer by circling in either (1) yes or (2) no. The classification of temporary housing is linked to the Kumamoto City ledger. The question was: “Please check all that apply to your current residence.” The options were (1) owned house, (2) houses for rent, (3) public housing, (4) public housing for disaster, (5) Hospitals and institutions, and (6) other.

#### Scope of relocation

For relocation (within or outside the community), participants were asked the following question: Is your current residence in the elementary community where you lived before the Kumamoto earthquake? Please circle the answer that applies. The answer choices were (1) in the same elementary community, (2) changed elementary community, and (3) did not know. This study was set up on an elementary school district basis, referring to the fact that previous studies have investigated the range of movement in residential areas or outside the city (Otsuka et al., 2023). Preventive care activities in Kumamoto City are conducted on a community basis (elementary school district basis) because information about community activities is mainly communicated through posts, circulars, verbal communication at meetings, and invitations among residents.

#### Daytime activities

Exercise Habits (1/w) included: “Do you walk or perform other exercises or household chores that replace exercise (cleaning, yard care, *etc.*) at least once a week? Please check all that apply”. The question was: “Do you perform household chores (cleaning, yard maintenance, *etc.*) to replace exercise and exercise such as walking at least once a week?” The answer choices were (1) Yes or (2) No. Questions on exercise habits were set up with reference to the Active Guide (Ministry of Health Labor Welfare of Japan, 2023) based on the Physical Activity Standards for Health Promotion 2013.

Regarding meal habits, please answer the question: Do you eat three meals per day? Please circle the answer that applies. The answer choices were (1) I eat and (2) I do not eat. The question about appetite was: “How is your appetite these days? Please circle the answer that applies.” The answer choices were as follows: (1) increased, (2) same as usual, and (3) decreased. The question about solitary eating was: “Who do you eat meals with most often? Please circle the answer that applies.” The answer choices were as follows: (1) eating alone, (2) eating with family, and (3) eating with friends, where “Eating alone” means solitary eating.

Social capital was ascertained through participation in community activities and the availability of social support (advisors). Participation in community activities was measured by asking: “Do you participate in events and social gatherings held in your community?” Respondents were asked to answer by circling in one of the following options: (1) I participate, (2) I do not participate, or (3) I do not know of any such information. The question was set because the same question was asked in an earlier recovery survey conducted in Kumamoto Prefecture; 62.1% of the respondents did not participate, and 4.4% did not have such information (Kumamoto Prefectural Government, 2021). Also, in Hikichi et al. (2017), which revealed social capital and physical and mental health, the question “Do you participate in community events?” and a yes/no response method was used.

The social support (advisor) question asked: “Who is the person you talk to about your problems?” The respondents were asked to circle in all the following options. The choices were: (1) family; (2) friend; (3) neighbors; (4) coworker; (5) district welfare commissioners; (6) hospital; (7) nursing care office; (8) city office; and (9) no one. For example, “(1) family” means to consult with one’s family. (2) to (9) are the same. Regarding social support, the roles of friends (Wu & Sheng, 2019), neighbors (Yoshiyuki & Kono, 2020), and welfare commissioners (Sugihara, 2018) are particularly important. In addition, medical institutions and nursing care offices are involved depending on the health conditions. These options were established based on the aforementioned criteria.

#### Insomnia

Insomnia was assessed using the Japanese version of the Athens Insomnia Scale (AIS), an 8-item insomnia rating scale developed by the WHO World Project on Sleep and Health (Okajima et al., 2013). Scores range from 0 to 24, with a score of six or higher being rated as “suspected insomnia” and is used for screening purposes (Kawasaki, Nishitani & Sakakibara, 2015).

#### Analysis method

**Figure 1:**
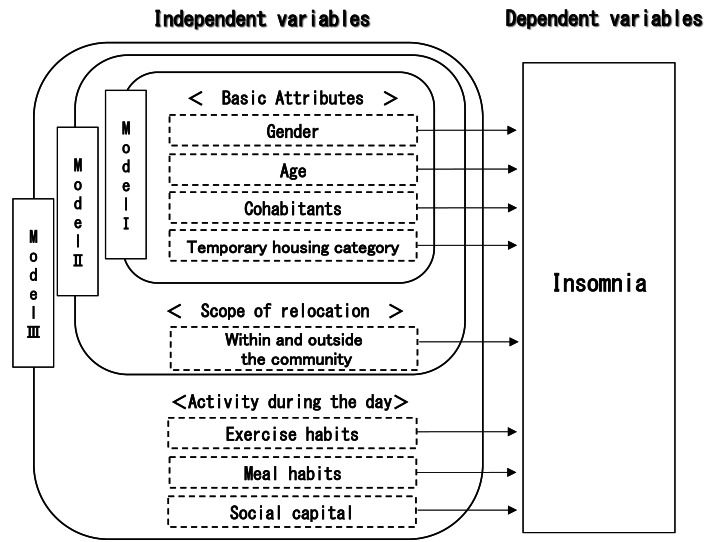
Analytical model. The framework of logistic regression analysis.

After calculating the basic statistics, a test of independence (X^2^ test) is performed for insomnia and basic attribute of relocation, and daytime activities. Next, binomial logistic regression analysis was conducted with insomnia as the dependent variable to examine the factors that influence insomnia. Three models were created, with the independent variables being basic attribute of relocation, and daytime activity (Fig. 1). Model I attempted to explain how insomnia is affected by basic attributes. Model II attempted to explain how insomnia was affected by adding the scope of relocation to Model I. Model III attempted to explain the impact of daytime activity on insomnia by adding daytime activity to Model II.

The independent variables are gender, age (whether the person falls into the category of 75 or older), presence of a roommate, temporary housing category, current residence, extent of relocation, exercise habits, dietary habits, and social capital. Dummy variables were used as independent variables, and the Variance Inflation Factor (VIF) was calculated using linear regression analysis to check for multicollinearity before conducting a dichotomous logistic regression analysis. Variables were selected using the forced entry method, and the two-sided statistical significance level was set at 0.05. The goodness of fit of the model will be performed with the X^2^ test and the Hosmer–Lemeshow test of the model. For the goodness of fit of the model, Negelkerke R^2^ values are calculated. Missing values for each variable were excluded. SPSS Statistics 29.0 for Windows was used as the statistical software.

## Results

Participants included 2,010 men (42.2%) and 2,748 women (57.8%). The average age of the participants was 75.36 ± 7.33 years (65–105), with 2,485 (52.2%) in the 65–74 age group and 2,273 (47.8%) in the 75+ age group. The basic attributes of the participants are listed in Table 1. Among the respondents, 3,473 (74.0%) live with a cohabitant. Most respondents (4,064 (85.4%)) lived in temporary housing. Regarding their current residence, 2,675 (56.7%) lived at home (owner-occupied), 1,024 (21.7%) lived in rented housing, and 768 (16.3%) lived in public housing. In addition, 1,494 (32.1%) participants moved out of the community.

**Table 1:** Demographics of the participants.

	*n* = 4,758	
	*n*	%
Gender		
Male	2,010	42.2
Female	2,748	57.8
Age		
Mean ± SD	4,758	75.36 ± 7.33
65–74	2,485	52.2
Over 75	2,273	47.8
Cohabitant		
Yes	3,473	74.0
No	1,218	26.0
Temporary housing category		
Prefabricated temporary housing	366	7.7
Temporary housing in the private sectors	4,064	85.4
Temporary housing in the public sectors	327	6.9
Current residence		
Owned house	2,675	56.7
Houses for rent	1,024	21.7
Public housing	768	16.3
Public housing for disaster	87	1.8
Hospitals and institutions	65	1.4
Other	96	2.0
Scope of relocation		
Within the community	3,160	67.9
Outside the community	1,494	32.1
Exercise habits		
Yes	3,460	75.9
No	1,097	24.1
Eat three meals a day		
Yes	3,955	84.8
No	710	15.2
Appetite		
Increase	172	3.7
Unchanged	3,451	74.1
Decrease	1,035	22.2
Meal partner		
Eating alone	1,467	32.3
Eating with family	3,018	66.4
Eating with friend	59	1.3
Community participation		
Participating community	1,331	28.9
No participating community	2,863	62.1
No getting information	415	9.0
Social support (Anyone)		
Consulting anyone	4,368	94.8
No one to consult	238	5.2
Social support (Family)		
Consulting family	3,601	78.1
No consultation with family	1,009	21.9
Social support (Friend)		
Consulting friend	1,657	36.0
No consultation with friend	2,949	64.0
Social support (Neighbors)		
Consulting neighbors	404	8.8
No consultation with neighbors	4,202	91.2
Social support (Coworker)		
Consulting coworker	190	4.1
No consultation with coworker	4,416	95.9
Social support (District welfare commissioners)		
Consulting district welfare commissioners	88	1.9
No consultation district welfare commissioners	4,518	98.1
Social support (Hospital)		
Consulting hospital	502	10.9
No consultation with hospital	4,104	89.1
Social support (Nursing care office)		
Consulting nursing care office	293	6.4
No consultation with nursing care office	4,313	93.6
Social support (City office)		
Consulting nursing city office	113	2.5
No consultation with city office	4,493	97.5
Social support (Other)		
Consulting other	242	5.3
No consultation with other	4,364	94.7

In addition, 3,460 (75.9%) participants were in the habit of exercising; 3,955 (84.8%) were in the habit of eating three meals per day; 1,035 (22.2%) reported a decrease in appetite; and 1,467 (32.3%) ate alone. Community activities were participated in by 1,331 (28.9%) participants. In contrast, 2,863 (62.1%) did not participate in community activities and 415 (9.0%) did not have such information. Regarding social support, 238 (5.2%) respondents had no one to talk to about social support. On the other hand, 3,601 (78.1%) answered that they consulted family members, 1,657 (36.0%) friend, 404 (8.8%) neighbors, 190 (4.1%) coworker, 88 (1.9%) disrict welfare commissioners, 502 (10.9%) hospital, 293 (6.4%) nursing care office, 113 (2.5%) city office, and 2.5%), and 242 (5.1%).

Table 2 shows the actual situation regarding insomnia; the mean AIS score was 5.01 ± 4.14 points. A total of 2,663 (62.4%) participants had a total score between 0 and 5, and 1,606 (37.6%) had a total score of 6 or more. Regarding sleep induction, 1,498 (32.7%) were “slightly delayed”, 595 (13.0%) were “markedly delayed ”, and 157 (3.4%) were “very delayed or did not sleep at all”. Regarding awakenings during the night, 1,236 (27.2%) were “minor problem”, 410 (9.0%) “were considerable problem”, and 50 (1.1%) were “serious problem or did not sleep all”. Regarding final awakening earlier than desired, 1,797 (39.6%) “a little earlier”, 521 (11.5%) “markedly earlier”, and 121 (2.7%) were “much earlier or did not sleep at all”. Regarding total sleep duration, 1,741 (37.6%) were “slightly insufficient”, 366 (7.9%) were “markedly insufficient”, and 59 (1.3%) were “very insufficient or did not sleep at all”. Regarding very insufficient or did not sleep at all, 1,972 (42.6%) were “slightly unsatisfactory”, 451 (9.7%) were “markedly unsatisfactory”, and 58 (1.3%) were “very unsatisfactory or did not sleep at all”. Regarding sense of well-being during the day, 1,074 (23.4%) were “Slightly decreased”, 199 (4.3%) were “markedly decreased”, and 43 (0.9%) were “very decreased”. As functioning during the day, 1,448 (31.4%) were ”slightly decreased”, 590 (12.8%) were “markedly decreased”, and 149 (3.2%) were “very decreased”. Sleepiness during the day was “mild” in 3,195 (69.5%), “considerable” in 492 (10.7%), and “intense” in 23 (0.5%).

**Table 2:** Prevalence rates of AIS (Athens Insomnia Scale).

	*n* = 4,269	
	*n*	%
AIS points		
Mean ± SD	4,269	5.01 ± 4.14
0–5	2,663	62.4
6–24	1,606	37.6
Sleep induction		
No problem	2,332	50.9
Slightly delayed	1,498	32.7
Markedly delayed	595	13.0
Very delayed or did not sleep all	157	3.4
Awakenings during the night		
No problem	2,847	62.7
Minor problem	1,236	27.2
Considerable problem	410	9.0
Serious problem or did not sleep all	50	1.1
Final awakening earlier than desired		
Not earlier	2,098	46.2
A little earlier	1,797	39.6
Markedly earlier	521	11.5
Much earlier or did not sleep at all	121	2.7
Total sleep duration		
Sufficient	2,461	53.2
Slightly insufficient	1,741	37.6
Markedly insufficient	366	7.9
Very insufficient or did not sleep at all	59	1.3
Very insufficient or did not sleep at all		
Satisfactory	2,150	46.4
Slightly unsatisfactory	1,972	42.6
Markedly unsatisfactory	451	9.7
Very unsatisfactory or did not sleep at all	58	1.3
Sence of well-being during the day		
Normal	3,272	71.3
Slightly decreased	1,074	23.4
Markedly decreased	199	4.3
Very decreased	43	0.9
Functioning during the day		
Normal	2,428	52.6
Slightly decreased	1,448	31.4
Markedly decreased	590	12.8
Very decreased	149	3.2
Sleepiness during the day		
None	888	19.3
Mild	3,195	69.5
Considerable	492	10.7
Intense	23	0.5

The results of the cross-tabulation of independent variables with and without insomnia are shown in Table 3. The variables significantly associated with the presence or absence of sleep were gender, age, cohabitant, type of temporary housing, current residence, scope of relocation, exercise habits, eat three meals a day, appetite, solitary meal, community participation, and social support. Regarding social support, significant differences were found in the presence or absence of anyone and family, coworker, hospital, nursing care office, city office, or other, while no significant differences were found for friend, neighbors, and district welfare commissioners.

**Table 3:** Cross-tabulation of AIS and independent variables.

	*n* = 4,269				
	Athens Insomnia Scale six points or more				
	Not applicable (*n* = 2,663)		Applicable (*n* = 1,606)		*p* value
	*n*	%	*n*	%	
Gender					0.004
Male	1,196	44.9	649	40.4	
Female	1,467	55.1	957	59.6	
Age					<0.001
65–74	1,501	56.4	801	49.9	
Over 75	1,162	43.6	805	50.1	
Cohabitant					<0.001
Yes	2,082	78.7	1,096	69.0	
No	565	21.3	492	31.0	
Temporary housing category					0.003
Prefabricated temporary housing	212	8.0	108	6.7	
Temporary housing in the private sectors	2,296	86.3	1,364	84.9	
Temporary housing in the public sectors	154	5.8	134	8.3	
Current residence					<0.001
Owned house	1,635	61.8	819	51.2	
Houses for rent	529	20.0	382	23.9	
Public housing	363	13.7	312	19.5	
Public housing for disaster	40	1.5	33	2.1	
Hospitals and institutions	31	1.2	13	0.8	
Other	46	1.7	40	2.5	
Scope of relocation					<0.001
Within the community	1,791	68.3	950	60.2	
Outside the community	832	31.7	627	39.8	
Exercise habits					<0.001
Yes	263	80.1	1,089	69.9	
No	514	19.9	468	30.1	
Eat three meals a day					<0.001
Yes	2,378	89.7	1,213	76.1	
No	274	10.3	380	23.9	
Appetite					<0.001
Yes	2,342	88.2	990	62.3	
No	312	11.8	598	37.7	
Eating alone					<0.001
Yes	659	25.6	629	40.5	
No	1,911	74.4	926	59.5	
Community participation					<0.001
Participating community	873	33.1	356	22.4	
No participating community	1,589	60.3	1,034	65.0	
No getting information	175	6.6	200	12.6	
Social support (Anyone)					<0.001
Consulting anyone	2,550	96.6	1,457	91.7	
No one to consult	89	3.4	132	8.3	
Social support (Family)					<0.001
Consulting family	2,206	83.6	1,120	70.4	
No consultation with family	434	16.4	470	29.6	
Social support (Friend)					0.442
Consulting friend	939	35.6	584	36.8	
No consultation with friend	1,700	64.4	1,005	63.2	
Social support (Neighbors)					0.485
Consulting neighbors	234	8.9	131	8.2	
No consultation with neighbors	2,405	91.1	1,458	91.8	
Social support (Coworker)					0.002
Consulting coworker	131	5.0	47	3.0	
No consultation with coworker	2,508	95.0	1,542	97.0	
Social support (District welfare commissioners)					0.691
Consulting district welfare commissioners	47	1.8	31	2.0	
No consultation district welfare commissioners	2,592	98.2	1,558	98	
Social support (Hospital)					<0.001
Consulting hospital	242	9.2	219	13.8	
No consultation with hospital	2,397	90.8	1,370	86.2	
Social support (Nursing care office)					<0.001
Consulting nursing care office	124	4.7	140	8.8	
No consultation with nursing care office	2,515	95.3	1,449	91.2	
Social support (City office)					<0.001
Consulting nursing city office	34	1.3	56	3.5	
No consultation with city office	2,605	98.7	1,533	96.5	
Social support (Other)					0.01
Consulting other	111	4.2	95	6.0	
No consultation with other	2,528	95.8	1,494	94.0	

**Notes.**

Pearson’s chi-square test, Fisher’s exact test.

To confirm multicollinearity, the VIF were calculated as 34.4, 23.6, 18, and 6 for the current type of residence (home, rental, and public housing, respectively). Therefore, current type of residence was removed from the independent variables in the binomial logistic regression analysis. The results of this analysis are shown in Table 4, with the presence or absence of insomnia as the dependent variable and basic attribute of change in residence, and daytime activities (exercise habits, meal habits, and social capital) as independent variables. Model I showed *p* < 0.001, Negelkerke R^2^ was 0.024, and the Hosmer–Lemeshow test showed *p* = 0.946. Model II showed *p* < 0.001, Negelkerke R^2^ showed 0.027 (*p* = 0.545 by the Hosmer–Lemeshow test). Model III also showed *p* < 0.001, Negelkerke R^2^ was 0.189, and *p* = 0.397 by the Hosmer–Lemeshow test. Therefore, both models fit. Furthermore, a comparison of Negelkerke R^2^ determined that the best model fit was Model III (0.189). Therefore, Model III was adopted for the binomial logistic regression analysis.

**Table 4:** Asssociation between the presence of insomnia and each independent variable.

	*n* = 4,234					
	Athens Insomnia Scale six points or more					
	model I		model II		model III	
	OR	95% CI	OR	95% CI	OR	95% CI
Gender (ref: Female)						
Male	0.88	0.77–0.99	0.87	0.77–0.99	0.76	0.66–0.89
Age (ref: 65–74)						
Over 75	1.25	1.10–1.42	1.27	1.12–1.44	1.17	1.004–1.35
Cohabitant (ref: Yes)						
No	1.58	1.37–1.83	1.45	1.25–1.68	0.77	0.60–0.99
Temporary housing category (ref: public housing)						
Prefabricated	0.64	0.46–0.90	0.69	0.49–0.97	0.77	0.52–1.13
Temporary housing rental	0.74	0.58–0.95	0.78	0.60–0.99	0.81	0.61–1.08
Scope of relocation (ref: Within the community)						
Outside the community			1.29	1.13–1.48	0.99	0.85–1.17
Exercise habits (ref: Yes)						
No					1.23	1.04–1.47
Eat three meals a day (ref: Yes)						
No					1.40	1.13–1.72
Appetite (ref: Yes)						
No					3.79	3.18–4.52
Eating alone (ref: Yes)						
No					1.60	1.27–2.02
Community participation (ref: Yes)						
No participating community					1.34	1.14–1.59
No getting information					1.94	1.47–2.55
Social support (ref: Yes)						
No one to consult					1.49	1.04–2.13
No consultation with family					1.49	1.21–1.84
No consultation with friend					0.80	0.68–0.94
No consultation with neighbors						0.83–1.40
No consultation with coworker					1.66	1.14–2.43
No consultation district welfare commissioners					1.18	0.69–2.04
No consultation with hospital					0.69	0.55–0.86
No consultation with nursing care office					0.64	0.48–0.87
No consultation with city office					0.53	0.32–0.89
No consultation with other					0.99	0.70–1.39
Negelkerke R2	0.024		0.027		0.189	

**Notes.**

OR Odds Ratio 95% CI 95% Confidence Interval ref reference

Binary logistic regression analysis.

Insomnia was associated with age 75+ (1.17, 1.004–1.35), no exercise habit (1.23, 1.04–1.47), not eating three times a day (1.40, 1.13–1.72), no appetite (3.79, 3.18–4.52), solitary meal (1.60, 1.27–2.02), no community participation (1.34, 1.14–1.59), no community information (1.94, 1.47–2.55), no one (1.49, 1.04–2.13), no family to consult (1.49, 1.21–1.84), and no colleagues to consult (1.66, 1.14–2.43).

## Discussion

### AIS scores of participants

The analysis covered 4,269 persons; the population of Kumamoto City in 2020 was 738,567, of whom 196,435 were aged 65 years or older. The 4,269 participants in this study comprised 2.2% of the total population.

In this study, 1,606 (37.6%) had an AIS total score of 6 or higher. In 2021, the Kumamoto Prefecture conducted a health survey of those affected by the Kumamoto earthquake in 17 municipalities that reside in temporary housing (Kumamoto Prefectural Government, 2021). The results showed that 66.6% responded that they were able to sleep and 28.4% said they were unable to sleep. In addition, in a 2017 Kumamoto Prefecture survey on health and dietary habits, 27.4% of respondents reported that they did not get enough rest from sleep (Kumamoto Prefectural Government, 2017). Furthermore, a study in older inpatients found insomnia in 19% (Ding, Qi & Sun, 2024), and a study of the prevalence of depression and insomnia among local older adults in Greece (Tsaras et al., 2021) found that 39.2% had an AIS total score of six or higher, with a mean ± SD of 4.08 ± 5.84 points. Kumamoto Prefecture is in the same region as the subject area of this study. The mean score for the elderly was 3.18 ± 2.47 for study subjects after the Great East Japan Earthquake (Iwasa et al., 2018). However, because the study included adults aged 18 years and older, we believe that the results show a higher rate of insomnia in the study population, which is a population of only older people. On the other hand, a study (Tsaras et al., 2021) conducted in the community-dwelling older adults in Greece showed almost the same percentage of insomnia. However, the mean AIS score was 0.93 points higher for the participants in this study. It is also higher than that of the elderly population after the Great East Japan Earthquake. It was suggesting that the population in this study is a relatively insomnia-prone group.

### Support for insomnia

In this study, the elderly who did not have an exercise habit were 1.23 times more likely to have insomnia than those who did. The elderly who did not eat three times were 1.40 times more likely to have insomnia than those who did. The elderly who did not have an appetite were 3.79 times more likely to have insomnia than those who did, strongly indicating that loss of appetite is a risk factor for insomnia. Rice et al. (2019) showed that changes in appetite and insomnia symptoms are present in depression with studies of adults. Duan et al. (2023) showed that sleep and circadian rhythms considerably influence the hormones involved in appetite regulation and energy metabolism. In this study, those who did not consume three meals per day had a higher risk of insomnia than those who did. This may be due to the dysfunction of energy metabolism caused by insomnia. Therefore, insomnia should be strongly suspected in individuals with no appetite, and support from local health and welfare professionals should be initiated at an early stage. In addition, those who did not exercise had a higher risk of insomnia than those who did. Siu et al. (2021) examined the effects of Tai Chi or exercise on sleep in older adults with insomnia and found that exercise and Tai Chi improved sleep, and the beneficial effects lasted for 24 months. The literature review of Moreno Reyes et al. (2020) also found that exercise should be the basis for insomnia. In other words, since exercise is effective in improving insomnia, the elderly who did not have an exercise habit were more likely to have insomnia than those who did in this study.

Next, those who did not participate in community activities were 1.34 times more likely to fall into the insomnia category than those who did, while friends, neighbors, and community members, who are human social resources in the community, were not significantly different from insomnia. Not participating in community activities is associated with physical inactivity (Kanamori, Ide-Okochi & Samiso, 2023). In addition, previous studies have shown that continuous physical activity, including exercise, requires a companion (Yarmohammadi et al., 2019) and a lack of social support from friends is associated with a lack of exercise habits in a study by Kanamori, Ide-Okochi & Samiso (2023). In addition, non-participation in community activities is associated with physical inactivity (Kanamori, Ide-Okochi & Samiso, 2023). Therefore, it was hypothesized that participation in community activities would increase social interactions and create connections with friends, neighbors, and community members, which could prevent or improve insomnia. However, no significant association was found in the present study. This study showed that approximately 38% of the respondents already had suspected insomnia, and those who consulted medical institutions, nursing care office, and city halls were more likely to have insomnia (see Table 3). In addition, the participants in this study were relatively prone to insomnia. It can be inferred that the subjects of this study were not at a stage where they could be helped by neighbors and district welfare commissioners but rather required appropriate support and treatment by professionals because they tended to have insomnia.

Furthermore, those who ate alone were 1.6 times more likely to be isolated; those who did not know about community activities were 1.94 times more likely to have no one to talk to; those who did not consult family members were 1.49 times more likely to be isolated; and those who did not consult colleagues were 1.66 times more likely to experience insomnia. These factors contribute to isolation and loneliness owing to a lack of human connections. Isolation and loneliness after a disaster have been associated with psychological distress, which is also strongly related to depression with insomnia as an initial symptom (Ide-Okochi et al., 2022). In addition, a decrease in the frequency of conversations among the elderly is significantly associated with loneliness (Ishimoto et al., 2024). Therefore, it was suggested that in order to prevent insomnia from a community health perspective, measures should be taken to prevent isolation by encouraging the elderly to gather together and engage in conversation.

Finally, while previous studies found that a larger area of relocation, such as moving outside the city, was associated with greater susceptibility to health problems (Sugawara, Tohmata & Tsuji, 2019; Tsuji, 2019). Model III in this study found no significant differences. This may be because this study asked only about moving within the same city and did not examine moves outside the city or prefecture, and the range of moves was relatively small. However, Model II and the *χ*2 test showed significant differences, and the possibility that people are more likely to fall into the category of insomnia if their residential community after relocation is different from their previous one cannot be denied. Therefore, the extent of relocation should be considered when providing support for insomnia.

These findings suggest the importance of the following two points in supporting insomnia: The first is to prevent isolation and loneliness by developing health activities based on informal support from the perspective of mutual assistance in the community. The second is early and appropriate intervention by formal support services, such as medical institutions, city halls, and care and welfare offices, for those suspected of having insomnia. Local health and medical welfare professionals are expected to make efforts to enhance both.

## Limitations

This study has a number of limitations.

The first limitation is due to the study design. Although temporal order is important in post-earthquake studies, this study was a cross-sectional study owing to the lack of longitudinal data. In addition, the limited explanatory power of the final model with an R^2^ of 0.189, the failure to distinguish between pre-existing and new cases of insomnia, and limited control for psychiatric comorbidities such as depression and PTSD limit the interpretation of causal relationships. In addition, the use of binomial logistic regression assumes linearity and independence of predictors but does not examine interactions between variables. This may increase the risk of Type I errors.

Second, this study omits trauma-related variables that are predictors of post-disaster mental health problems. This may confound the relationship between relocation and insomnia and may weaken the validity of the findings.

Third, there is the issue of self-reported data and measurement. Considering that AIS alone is insufficient for assessing the severity and functional impairment of insomnia, it is desirable to consider complementary assessment methods in accordance with study objectives in the future. In addition, social capital and exercise habits are operationalized in a simplistic manner, and so nuances such as frequency, intensity, and quality of interactions may be overlooked. Additionally, it may have been difficult to accurately answer some of the questions. For example, the question, “Do you perform household chores in place of exercise?” could not have been answered by those who do perform household chores but not in place of exercise. Additionally, the question, “How is your appetite these days?” does not specify a comparison timepoint, and so the responses may have varied.

Fourth, there is a selection bias. The sample was limited to those who had left temporary housing, excluding those who were still in temporary housing and those who chose not to participate. This may have resulted in a small number of responses from very vulnerable groups and may have biased the results toward healthier respondents.

Fifth, this study set the scope of relocation at the elementary school district level, in line with the activity base of Kumamoto City, the affected area. Therefore, there may be limitations in generalization.

## Conclusions

This study showed that daytime activities such as exercise habits, meal habits, and social capital, as well as the extent of relocation, were significantly associated with insomnia among the elderly affected by the Kumamoto earthquake.

Therefore, support for insomnia requires both informal supports to prevent isolation based on mutual community support and formal support for those suspected of insomnia. We hope that study will continue on effective ways of providing support in the future. It is assumed that individual and group support for subjects will be realized through collaboration between the two, mainly by health and medical welfare professionals in the community.

##  Supplemental Information

10.7717/peerj.20584/supp-1 Supplemental Information 1 Raw data

10.7717/peerj.20584/supp-2 Supplemental Information 2 Categorical data codes

## Additional Information and Declarations

### Data Availability

The following information was supplied regarding data availability: The raw measurements are available in the Supplementary Files.
